# Utilization of Pharmacokinetic/Pharmacodynamic Modeling in Pharmacoepidemiological Studies: A Systematic Review on Antiarrhythmic and Glucose-Lowering Medicines

**DOI:** 10.3389/fphar.2022.908538

**Published:** 2022-06-20

**Authors:** Soroush Mohammadi Jouabadi, Mitra Nekouei Shahraki, Payam Peymani, Bruno H. Stricker, Fariba Ahmadizar

**Affiliations:** ^1^ Department of Epidemiology, Department of Internal Medicine, Erasmus MC University Medical Center, Rotterdam, Netherlands; ^2^ Division of Pharmacology, Department of Internal Medicine, Erasmus MC University Medical Center, Rotterdam, Netherlands; ^3^ Julius Global Health, University Medical Center Utrecht, Utrecht, Netherlands

**Keywords:** pharmacodynamics (PD), pharmacokinetics, pharmacoepidemiology, glucose-lowering agents, antiarrhythmic, PK/PD modeling

## Abstract

**Introduction:** In human pharmacology, there are two important scientific branches: clinical pharmacology and pharmacoepidemiology. Pharmacokinetic/pharmacodynamic (PK/PD) modeling is important in preclinical studies and randomized control trials. However, it is rarely used in pharmacoepidemiological studies on the effectiveness and medication safety where the target population is heterogeneous and followed for longer periods. The objective of this literature review was to investigate how far PK/PD modeling is utilized in observational studies on glucose-lowering and antiarrhythmic drugs.

**Method:** A systematic literature search of MEDLINE, Embase, and Web of Science was conducted from January 2010 to 21 February 2020. To calculate the utilization of PK/PD modeling in observational studies, we followed two search strategies. In the first strategy, we screened a 1% random set from 95,672 studies on glucose-lowering and antiarrhythmic drugs on inclusion criteria. In the second strategy, we evaluated the percentage of studies in which PK/PD modeling techniques were utilized. Subsequently, we divided the total number of included studies in the second search strategy by the total number of eligible studies in the first search strategy.

**Results:** The comprehensive search of databases and the manual search of included references yielded a total of 29 studies included in the qualitative synthesis of our systematic review. Nearly all 29 studies had utilized a PK model, whereas only two studies developed a PD model to evaluate the effectiveness of medications. In total, 16 out of 29 studies (55.1%) used a PK/PD model in the observational setting to study effect modification. The utilization of PK/PD modeling in observational studies was calculated as 0.42%.

**Conclusion:** PK/PD modeling techniques were substantially underutilized in observational studies of antiarrhythmic and glucose-lowering drugs during the past decade.

## 1 Introduction

There are two important scientific branches in human pharmacology, namely, clinical pharmacology with pharmacokinetic/pharmacodynamic modeling among small patient groups and pharmacoepidemiology with observational studies with effect modeling of medications in large populations or healthcare databases. The question arises as to how far these branches interact with a mutual exchange of information and expertise to attain some synergy by combining and integrating methodology and scientific output. Because in clinical pharmacology, the individual is the study subject, whereas pharmacoepidemiology focuses on effects in study populations, it seems that there is much to be gained where an overlap exists.

Pharmacokinetics (PK) generally describes the time–concentration relationship of the drug in body fluids and tissues in order to investigate the effect of the body on drugs to learn how drugs are absorbed, metabolized, and excreted. PK studies help identify recommendable dosage schemes, as well as predict different toxicity profiles, maximum drug concentration, and food/drug–drug interactions (Derendorf et al., 2000; Charles, 2014; Tamargo et al., 2015; Garralda et al., 2017). Pharmacodynamics (PD) refers to identifying the potential effects of a drug on the body and its physiology, including therapeutic effects and adverse effects. PD studies investigate the relationship between drug concentration at the receptor site and the receptor’s response to different drug concentrations. PK and PD demonstrate the link between the dose–response curves of a particular drug (Derendorf et al., 2000; Felmlee et al., 2012; Garralda et al., 2017). Physiologically based pharmacokinetic (PBPK) modeling predicts both systemic and tissue concentration–time profiles in individuals (Tan et al., 2019) as the basis of individual pharmacodynamic effects. Clinical pharmacologists are constantly moving this forward from individuals to groups with population-based PK/PD studies to evaluate the intra- and inter-individual variability, for instance, variability by gender (Harris et al., 1995), race (Johnson, 2000), or body mass index (BMI) (Hanley et al., 2010) in a specific population and to investigate its associated effects on the PK profile of the medication and its therapeutic response and dosing optimization (Vinks, 2002; Mould and Upton, 2013; Charles, 2014; Bensalem and Ternant, 2020). This information should be a basis for individualized therapy and developing a proper dosage regimen when a favorable therapeutic target goal is set (Vinks, 2002; Jayachandran et al., 2015). Utilization of PK/PD modeling is not only warranted in preclinical studies and randomized control trials (RCTs) as a part of new drug applications, but also PK/PD modeling data are essential for widespread and large-scale use in heterogeneous populations such as the elderly and children and long-term follow-up. The therapeutic window of antiarrhythmic drugs is narrow, and hence, a perfect target for PK/PD studies as small changes in the dosage will lead to a considerable PD alteration (Blix et al., 2010; Tamargo et al., 2015). Furthermore, PK/PD modeling is not only important for drugs with a narrow therapeutic window but also for other medications with a wider index. Glucose-lowering drugs have large therapeutic indexes and are safe. However, diabetic patients may encounter several complications during their life, and therefore, it is important to optimize and individualize the treatment for these patients throughout PK/PD modeling (Landersdorfer and Jusko, 2008). In observational settings, the effects of drugs are studied on a population-based scale. Hence, one might expect that population PK/PD modeling is an important part of pharmacoepidemiology. It should be possible to integrate both branches of human pharmacology to overcome the discrepancy between clinical and real-world medication outcomes. Unfortunately, although applying PK/PD modeling is possible in observational/pharmacoepidemiological studies, it seems that it was only scantily performed until now (Ette et al., 2003; Herland et al., 2005; Charles, 2014; Standing, 2017). Therefore, the objective of this literature review was to investigate how far PK/PD modeling has been utilized in pharmacoepidemiological studies during the past decade and to identify the different types of models and their specific objectives.

## 2 Methods

A systematic literature review was performed according to the most recently updated PRISMA (Preferred Reporting Items for Systematic Review and Meta-Analysis) guideline (Page et al., 2021). The study was performed in a work package as part of an EU-sponsored IMI-project [grant agreement 116,030], and the study focused on antiarrhythmic and glucose-lowering drugs.

### 2.1 Search Strategy

#### 2.1.1 Search Strategy 1

A literature search of MEDLINE, Embase, and Web of Science was conducted from January 2010 to 21 February 2021 based on all MeSH terms and keywords, including but not limited to [observational studies] AND [glucose-lowering agents OR antiarrhythmic drug]. According to a large number of retrieved studies with this strategy and consequently impractical screening, we followed a sampling method. We selected 1% (956 studies) of the total number of studies (95,672) that were selected using the ENDNOTE–Excel random sampling method (Wanner, 2017). After that, by screening the 956 studies in the random set, the number of studies fulfilling the inclusion criteria was identified. Eventually, the proportion of true-positive identified studies out of 956 was used to estimate the number of all observational studies on glucose-lowering medications and antiarrhythmic drugs within the last 10 years (denominator).

#### 2.1.2 Search Strategy 2

To evaluate the percentage of PK/PD modeling utilization among the aforementioned observational studies, MeSH terms describing a PK/PD model were added to the previous search query, including ([observational studies] AND [glucose-lowering agents OR antiarrhythmic drug] AND [Pharmacokinetic OR Pharmacodynamics], (Supplementary Table) (numerator to calculate the utilization of PK/PD modeling). The summary of both search strategies is presented in Table1. In addition, the references of the included studies were manually searched for any further appropriate studies. The prevalence of utilizing PK/PD modeling in observational studies was calculated by dividing the numerator by the denominator.

**TABLE 1:** Eligibility criteria.

Search strategy 1		Search strategy 2	
Inclusion criteria	Exclusion criteria	Inclusion criteria	Exclusion criteria
Observational study	Clinical trials, case reports, case series, editorial, abstracts, and commentary.	Observational study	Clinical trials, case reports, case series, editorial, abstracts, and commentary
Glucose-lowering medications or antiarrhythmic medications	Any other medications rather than these two groups or are not according to the ATC classification	Glucose-lowering medications or antiarrhythmic medications	Any other medications rather than these two groups or are not according to the ATC classification
Human study	Animal/experimental studies	Human study	Animal/experimental studies
Between 2010–2020	Out of this range	Between 2010–2020	Out of this time-interval
NA	NA	Applying a PK/PD model	Not applying a PK/PD model

Abbreviations: NA, Not Applicable; ATC, Anatomical Therapeutic Chemical Classification System; PK/PD, Pharmacokinetic/Pharmacodynamic.

### 2.2 Data Screening and Abstraction

Two researchers (SMJ and PP) independently screened articles by their titles and abstracts, and any discrepancies were solved by consensus. Afterward, articles were screened by their full text in the second eligibility assessment round and then highlighted as eligible if they had fulfilled the inclusion criteria. Any disagreement in this step was resolved by a third researcher (FA). Furthermore, the data of eligible studies were extracted by study design, study year, type of PK/PD model, effect modifiers, medication name, and PK/PD parameters using a designated form.

### 2.3 Eligibility Criteria

The most recent 10-year period [2010–2020] was applied. Inclusion criteria were observational studies (cohorts, cross-sectional, and case–control studies) and human studies on glucose-lowering or antiarrhythmic medications with no language restriction. For the second search strategy, additionally, we included a study if a PK/PD model was developed or utilized (Table 1).

## 3 Results

### 3.1 Search Strategy 1

**FIGURE 1:**
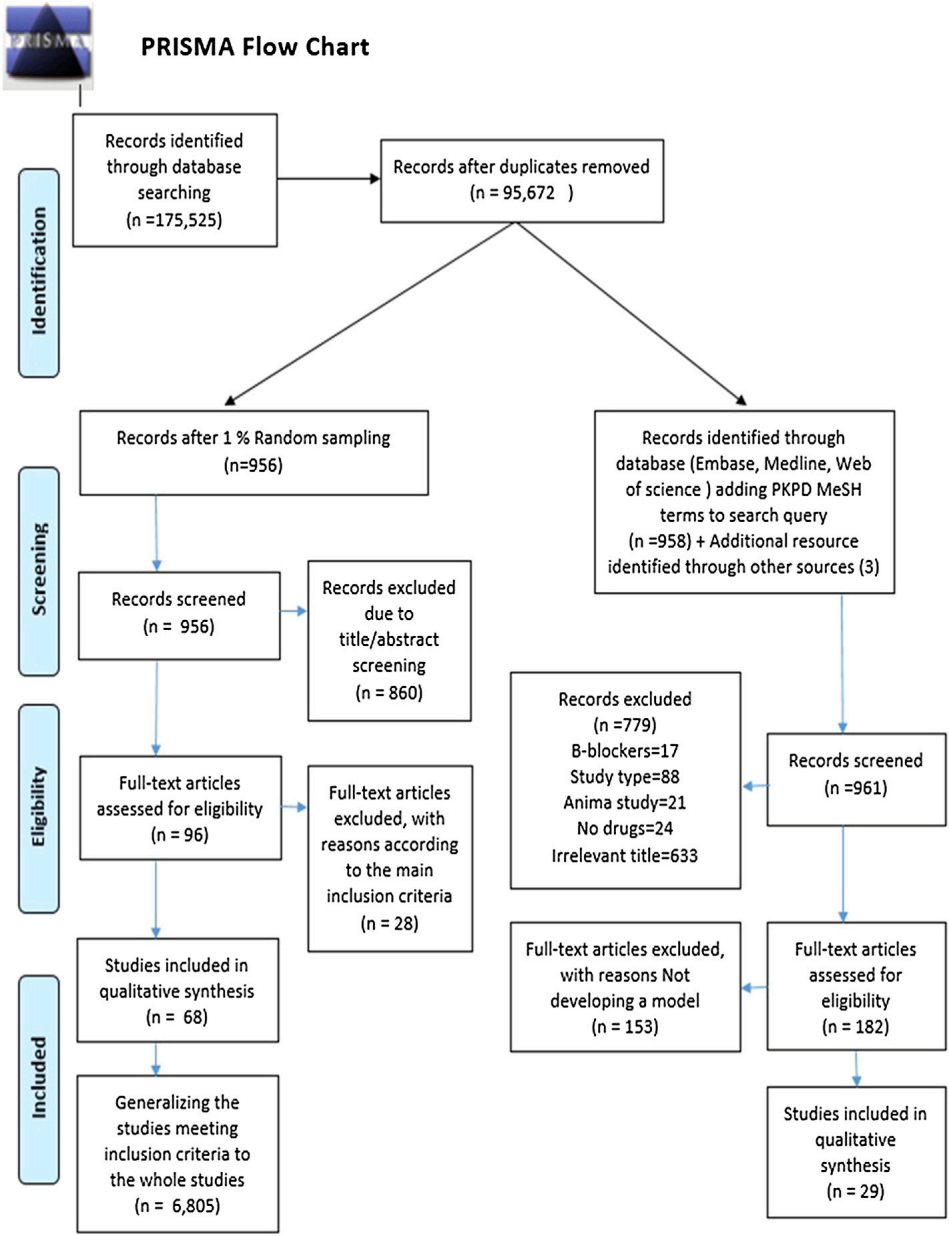
PRISMA flow chart.

In total, 95,672 records were identified within the literature search. After establishing a 1% random set, 956 studies were screened for their title/abstract, and 860 were eliminated according to the inclusion criteria. The full text of the remaining 96 studies was assessed for eligibility resulting in 68 qualified articles meeting our inclusion criteria. Consequently, by generalizing the 1% sampling to the entire identified records, 6,805 studies fulfilled our aforementioned criteria. This result indicates that approximately 6,805 observational studies (of any type) investigated glucose-lowering and antiarrhythmic medications between 2010 and 2020. The retrieved number was used as the denominator of the utilization proportion. The PRISMA flowchart of the randomization and selection procedure is depicted in Figure 1.

### 3.2 Search Strategy 2

The comprehensive search of databases, in addition to the manual search of included references, yielded a total of 1,256 citations by limiting the previous result (95,672 studies) to the PK/PD mesh terms. In contrast, 1,074 studies were excluded after the title/abstract screening. Of the 182 remaining studies undergoing full-text screening, 29 were included in the qualitative synthesis of our systematic review. Other studies were excluded because they did not apply a PK/PD model—these 29 pharmacometric studies were used as the numerator of the utilization proportion. The process of study identification and eligibility is shown in Figure 1.

**FIGURE 2:**
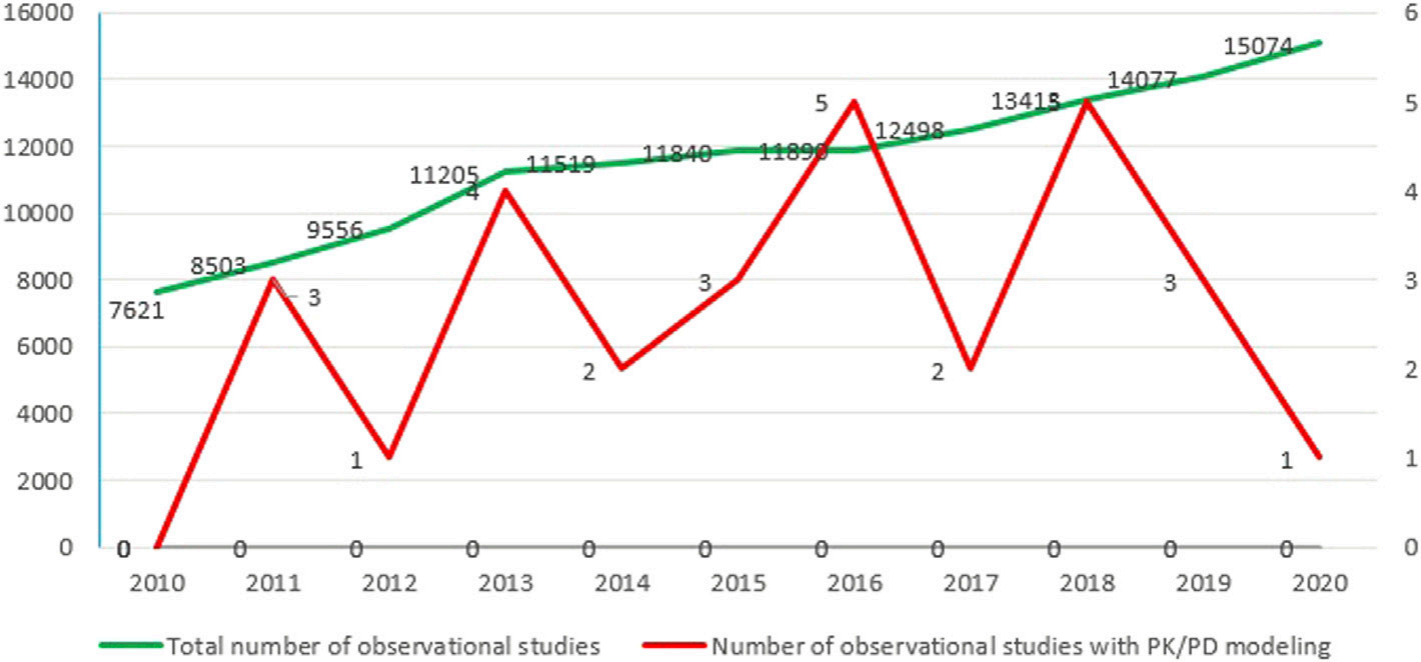
Decade of trend in the number of observational studies with PK/PD modeling.

By dividing the retrieved numerator by the denominator, the utilization proportion of PK/PD modeling in observational studies was calculated as 0.42% (Figure 2).

**FIGURE 3:**
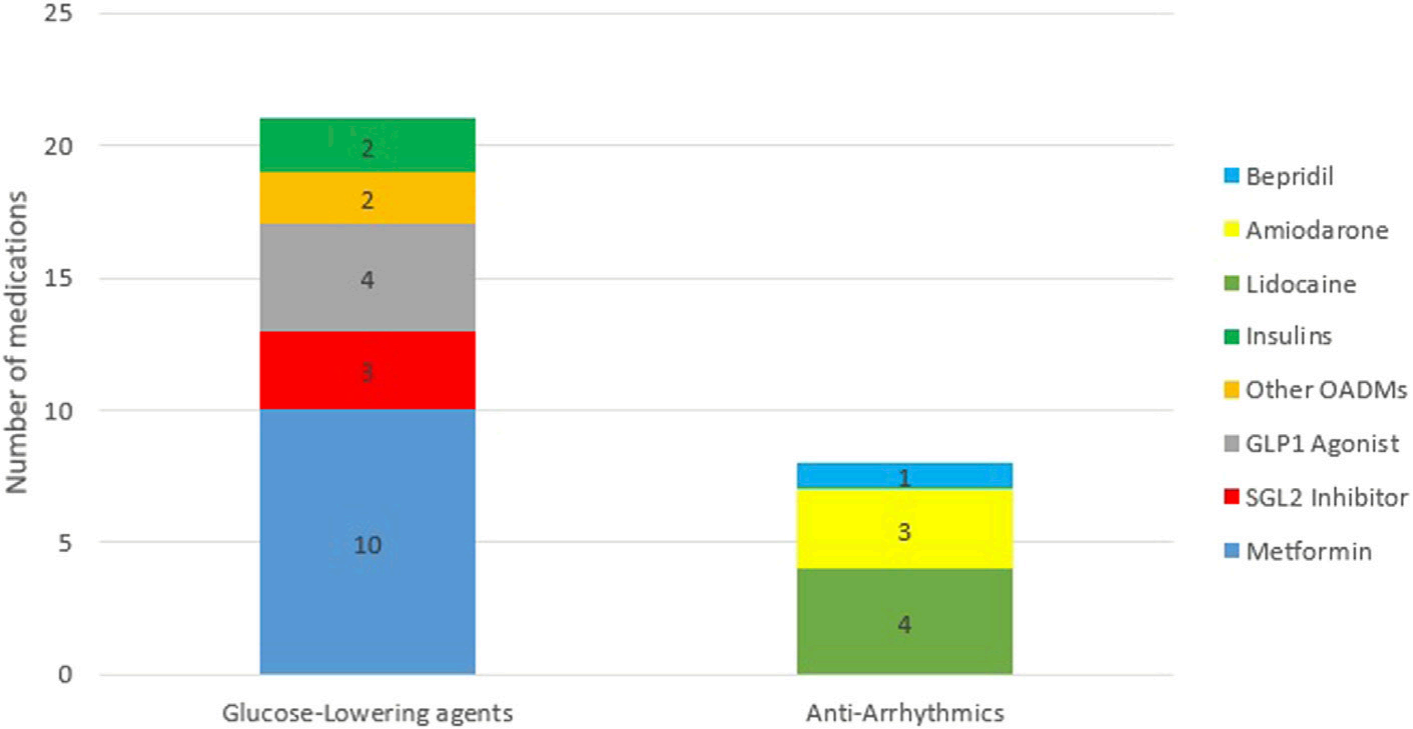
Frequency distribution of medications. Abbreviations: OADMs, Oral Anti-diabetic Medications; GLP1 Agonist, glucagon-like peptide 1 Agonist; SGL2 Inhibitors, Sodium-glucose Cotransporter-2 Inhibitors.

While the majority of the 29 included studies (21) investigated glucose-lowering medications, eight studies pertained to antiarrhythmic medications (Araki et al., 2011; Hsu et al., 2011; van den Broek et al., 2011; Shiga et al., 2013; Salem et al., 2016; Bursi et al., 2017; Dallefeld et al., 2018; Riff et al., 2018). As shown in Figure 3, nearly half (48%) of the studies on glucose-lowering drugs focused on metformin and half of the studies on antiarrhythmic drugs concerned lidocaine.

Nearly all studies had utilized a PK model. In contrast, only two studies by Salem et al. (2016) and Stringer et al. (2015) developed a PD model to evaluate the effect of medications in a cohort and case–control study design, respectively. Of the 182 studies included for full-text screening, twenty studies focused on antiarrhythmic, while eight (40%) applied PK/PD models. In total, 21 (14.5%) out of 144 studies on glucose-lowering medications applied PK/PD models.

**FIGURE 4:**
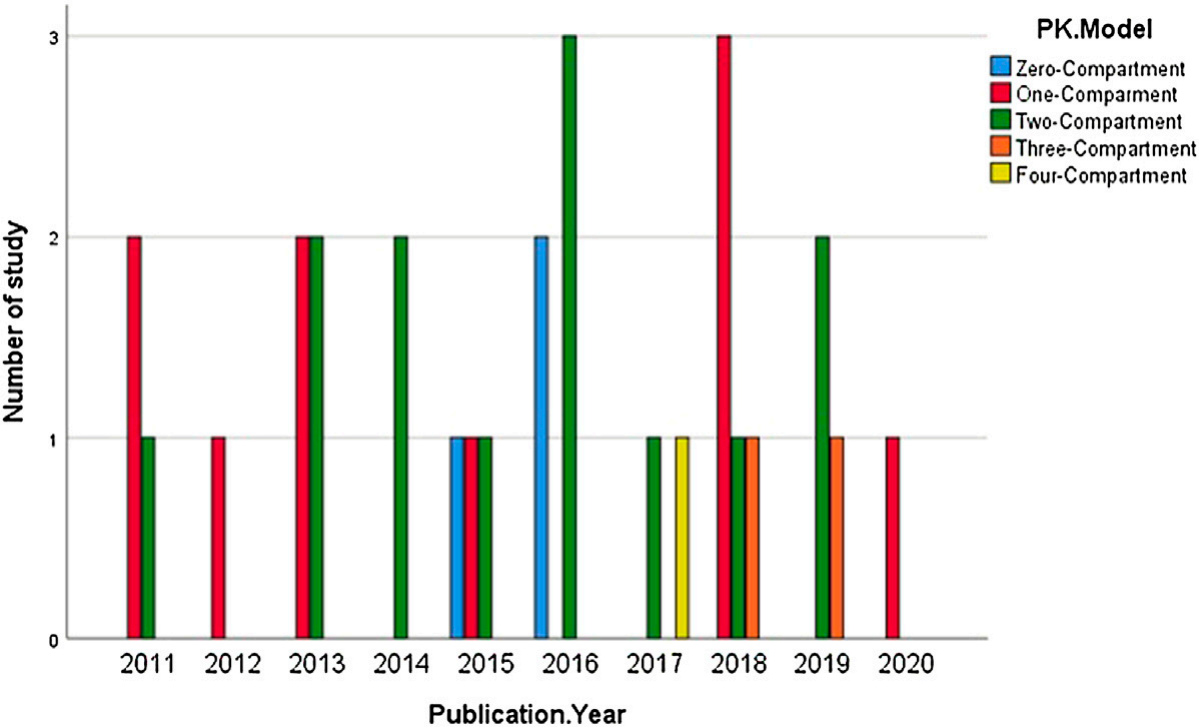
Distribution of different types of PK models.

Also, 11 out of 27 studies used a two-compartmental PK model to describe the action of medications. Contrarily, only 1 study (Bursi et al., 2017) developed a model using four-compartmental dispositions (Figure 4).

Also, 16 out of 29 studies (55.1%) used a PK/PD model in the observational setting with the aim of treatment individualization according to different patients’ clinical characteristics and deriving the safest and the most effective dose of the treatment, not only in a small group of patients but also in a large population. A total of 9 studies have evaluated the effect of population demographics (age, sex, race, and BMI, etc.) and 4 articles evaluated the outcome of genetic variation by applying a PK/PD model. The characteristics and purpose of developing a PK/PD model for each included study have been described in Table2.

**TABLE 2:** Characteristics of included studies.

Author and publication year	PK/PD model and type of model	Purpose of applying or developing a PK/PD model	Effect modifier
Araki et al. (2011)	PK model	To investigate the role of patient characteristics in estimating doses of amiodarone using routine therapeutic drug monitoring data and to improve the predictive performance of the population pharmacokinetic parameters in a high-concentration area	BMI, daily dosage, and duration of dosing
	One-compartmental		
Hsu et al. (2011)	PK model	To determine the pharmacokinetics of lidocaine in a 48-h infusion in patients undergoing cardiac surgery with cardiopulmonary bypass	Weight and diabetes mellitus status
	Two-compartmental		
van den Broek et al. (2011)	PK model	To develop an optimized dosing regimen for lidocaine in preterm and term neonates	Body size and physiologic maturation
	One-compartmental		
Shiga et al. (2013)	PK model	To evaluate the contributing factors to changes in the dose–concentration relationship of bepridil and the risk factors for excessive QT prolongation in patients with paroxysmal or persistent AF	NA
	One-compartmental		
Salem et al. (2016)	PD model	To investigate the effective doses and covariates influencing amiodarone efficacy	Pre-treatment with amiodarone, catecholamine infusion, and magnesium loading
Bursi et al. (2017)	PK model	Further insights into the evaluation of the pharmacokinetic properties of lidocaine and its metabolites to assess its safety	BMI, body fat, smoking, creatinine level, and AST/ALT level
	Four-compartmental		
Dallefeld et al. (2018)	PK model	Characterizing amiodarone disposition in children	NA
	Three-compartmental		
Riff et al. (2018)	PK model	To describe lidocaine pharmacokinetics in older women undergoing breast cancer surgery after TLA and to explore the risk of the toxicity of this technique	NA
	One-compartmental		
Bardin et al. (2012)	PK model	To develop a population pharmacokinetic model for metformin in patients with type 2 diabetes mellitus over a wide range of body weights and evaluate different size descriptors more specifically	NA
	One-compartmental		
Haidar et al. (2013)	PK model	To estimate pharmacokinetic parameters of insulin and glucagon during closed-loop operation	NA
	Two-compartmental		
Riggs et al. (2013)	PK model	To develop a population PK model from phase I and II data to estimate the effects of covariates, such as demographics, patient habits, and laboratory values, which may explain variability in empagliflozin PK parameters	Race and total protein
	Two-compartmental		
Yoon et al. (2013)	PK model	To assess the effect of genetic polymorphisms in organic cation transporters (OCTs) on the population pharmacokinetics of metformin	Liver function and genetic polymorphism
	One-compartmental		
Gertz et al. (2014)	PK model	To investigate the effect of OATP1B1 genotype as a covariate on repaglinide pharmacokinetics and drug–drug interaction (DDI) risk	Genetic polymorphism
	Two compartmental		
Goswami et al. (2014)	PK model	To investigate the effect of prioritized transcription factor variants on the systemic plasma levels of metformin in both patients and healthy subjects	NA
	Two-compartmental		
Duong et al. (2015)	PK model	To investigate and compare the clearance of metformin in indigenous and non-Indigenous patients with T2DM	NA
	Two-compartmental		
Petri et al. (2015)	PK model	To compare the pharmacokinetics of liraglutide in children and adolescents and to determine whether the adult dosing regimen is appropriate for future clinical trials in this pediatric population	NA
	One-compartmental		
Stringer et al. (2015)	PD model	To enhance the understanding of the treatment and time-course effects on FPG and HbA1c and to develop a model to enable the simulation for both groups and compare the longer-term glycemic durability	BMI, number of non- thiazolidinedione medications, baseline FPG, and HbA1C
Chitnis et al. (2016)	PK model	To evaluate whether country-sourced metformin is a significant covariate for different bioavailability	BMI, country-sourced metformin, and race
	Two-compartmental		
Geiser et al. (2016)	PK model	To characterize the pharmacokinetics of dulaglutide, estimate the associated variability in the target patient population, and evaluate potential intrinsic and extrinsic factors that may significantly influence dulaglutide pharmacokinetics	Different dose, BMI, weight, race, and smoking status
	Two-compartmental		
Hoeben et al. (2016)	PK model	To develop a population-based PK model that adequately describes the PK of canagliflozin after oral administration in healthy volunteers and patients with T2DM and to evaluate the effects of ‘volunteer’ demographic characteristics and other covariates on PK parameters of canagliflozin	BMI, eGFR, and diabetes
	Two-compartmental		
Santoro et al. (2016)	PK model	To develop and validate limited sampling strategy (LSS) models to predict the area under metformin’s plasma concentration–time curve (AUC)	NA
	Non-compartmental model		
Duong et al. (2017)	PK model	To investigate the proportion of metformin cleared by the kidneys (CLR), the proportion of the drug not cleared by the kidneys (non-renal clearance of metformin, CLNR/F), and the drug exposure (AUC0–12,AUC0–24) of metformin in a large sample of patients with varying degrees of renal function	NA
	Two-compartmental		
Petri et al. (2018)	PK model	To evaluate the effect of sex, age, race, ethnicity, body weight, renal function, maintenance dose level used, and injection site chosen on the individual average steady-state plasma concentrations of semaglutide	Race, ethnicity, renal function, and weight
	One-compartmental		
Ceacareanu et al. (2018)	PK model	To predict metformin’s clearance in acute myelogenous leukemia (AML) population	NA
	Two-compartmental		
Dissanayake et al. (2018)	PK model	To assess the impact of kidney function on single-dose metformin PK profiles	Renal function
	One-compartmental		
Overgaard et al. (2019)	PK model	To investigate the impact on HbA1c and body weight on switching to semaglutide from other GLP-1RAs (liraglutide, dulaglutide, and exenatide ER) and to analyze different dose-escalation algorithms depending on the PK of each GLP-1RA	HbA1C and weight
	Several different compartment models		
Sokolov et al. (2019)	PK model	To assess the dapagliflozin exposure–response relationship in Japanese and non-Japanese patients with type 1 diabetes mellitus (T1DM) and investigate if a dose adjustment is required in Japanese patients	NA
	Two compartmental		
van Noorden et al. (2019)	PK model	To develop a first model of insulin detemir and its unique action and validate it against existing data in the literature	NA
	Three-compartmental		
Li et al. (2020)	PK model	To determine the dosing regimen of metformin in patients with T2DM. It was undertaken to estimate the pharmacokinetic parameters of metformin and to evaluate the impact of demographic and genetic polymorphism factors on metformin disposition	eGFR, genetic polymorphism, and BMI
	One-compartmental		

PK: pharmacokinetics, PD: pharamacodynamics, BMI: body mass index, T2DM: type 2 diabetes mellitus.

## 4 Discussion

This analysis of almost 100,000 publications on glucose-lowering and antiarrhythmic drugs over 10 years showed that PK/PD modeling studies and observational studies are in two distinct worlds. This is somewhat disappointing because both clinical pharmacology and pharmacoepidemiology intend to study drug effects in humans, and many of the pharmacokinetic and dynamic determinants are important effect modifiers in pharmacoepidemiological studies. This systematic review is the first appraisal of PK/PD utilization in observational studies, to the best of our knowledge. We showed that, in the past decade, the application of PK/PD modeling has been substantially underutilized in pharmacoepidemiological studies. This is unfortunate because PK/PD modeling gives an insight into important effect modifiers influencing drug metabolism and efficacy and would facilitate risk estimates in pharmacoepidemiology in subgroups and high-risk strata. By taking advantage of PK/PD modeling in large populations, the effects of different covariates (confounders or effect modifiers) on the pharmacokinetic profile of specific medications could be straightforwardly predicted in the real-world setting, which also will be of value for personalized medicine concepts (Boyko, 2013).

Our study showed no obvious growing trend in PK/PD modeling utilization in the past 10 years. The different scientific roots of clinical pharmacology and pharmacoepidemiology might partly explain this. PK/PD modeling was developed in animal studies and human volunteers to investigate drugs’ pharmacokinetics and pharmacodynamics effect on human biology. Subsequent PK/PD modeling in clinical trials in homogenous patient groups to optimize dosing schedules was nevertheless considered of limited value in predicting the response in large and heterogeneous populations (Vinks, 2002). Although pharmacoepidemiology started mainly quantifying adverse reactions and their risk factors after marketing, the increasing size of healthcare databases nowadays stimulates comparative studies on effectiveness. Unfortunately, such databases rarely include information that can be used for personalized fine-tuning of results within various risk groups (Van Boxtel and Wang, 1997). For instance, information on body mass index or smoking is often missing, while such determinants are often relevant. Another important reason for this underutilization may be because blood sampling and in-patient observations are often not feasible in a pharmacoepidemiological setting. In clinical trials, detailed patient information is more easily obtained. Consequently, observational studies have some limitations which can be avoided in randomized control trials to a great extent by complete data collection.

Our review was limited to two pharmacotherapeutic groups, that is, glucose-lowering and antiarrhythmic drugs as representative agents of medications with a wide and narrow therapeutic window, respectively. Due to the striking global prevalence of diabetes (6.28% in 2017) in comparison to cardiac arrhythmia (0.51% in 2017) (Khan et al., 2020; Lippi et al., 2021), the use of glucose-lowering agents is higher than that of antiarrhythmic drugs. In line with this, the selection of articles from the current study sample also shows that the total number of studies on glucose-lowering medications (21 studies) is higher than that on antiarrhythmic (8 studies). However, in addition to this lower number, our literature search suggests that more effort has been put into applying PK/PD models for antiarrhythmic drugs. An important reason for this higher PK/PD modeling yield in studies on antiarrhythmic drugs is their narrower therapeutic window (Muller and Milton, 2012). After all, the narrower the therapeutic window, the higher the probability of toxicity or adverse effect, and therefore, more PK/PD profile monitoring is required (Tamargo et al., 2015). The study by Caruso et al. (2014) also confirmed the importance of applying the PK/PD model to studies on antiarrhythmic drugs to enable a more accurate prediction of the medication’s clinical effects.

PK/PD modeling is highly encouraged in other vital therapeutic processes, not only in medications with a narrower therapeutic index but also in pediatric clinical research (De Cock et al., 2011). Furthermore, De Cock et al. explained the pivotal role of PK/PD modeling in studies on children to predict the possible therapeutic failure and occurrence of adverse effects or death.

Our systematic review observed that most studies developed the PK/PD model to evaluate the effect of different covariates on therapeutic outcomes in small- or medium-sized populations. Our result is in line with a previous study (Standing, 2017) that showed that using a PK/PD model can improve the approach to personalized medicine where the effect of variables such as age, sex, BMI, and genetic variation could be adjusted.

It should be taken into account that most included studies applied a PK/PD model to the secondary data. Secondary data are data which cannot be traced back to the level of individual cases of statistical units. These data sources can be retrieved by collecting the primary data from trials with matching population characteristics. For instance, the data had been collected for a particular purpose/research question but again utilized to answer another research question. The utilization of secondary data is more practical than collecting primary data from large populations, and it is also more economical (Schneeweiss and Avorn, 2005; Suissa and Garbe, 2007). Moreover, with the recent advances in electronic healthcare databases, pharmacoepidemiologists have easier access to these data to boost their investigations on medications’ effectiveness and individualized dosage optimization (Hennessy, 2006). For instance, clinical trial data on the incidence of cardiovascular endpoints in age-, sex-, and BMI-stratified analyses matched to observational studies could improve the quality of effectiveness studies in pharmacoepidemiology. In the future, individual pharmacogenetic passports can be used to tailor individual pharmacotherapy, especially if genetic analyses in responders/non-responders would become a common practice in drug trials. PK/PD modeling thereby will play a crucial role in the efficacy and safety assessment of recently developed medications and optimizing treatment. The synergy between clinical pharmacology and pharmacoepidemiology could be substantially improved if more effect- and risk-stratification in clinical trials would occur to investigate which subgroups’ response and toxicity are the highest. In this way, recognizing determinants of drug response and toxicity would provide important information on effect modifiers available in observational datasets. This would improve the validity and efficiency of real-life effectiveness studies.

## 5 Conclusion

Overall, there is a lack of synergy between clinical pharmacology and pharmacoepidemiology, especially large-sized observational studies make only limited use of information on effect modification from PK/PD studies. On the other hand, clinical trials might pay more attention to risk stratification, for instance, by pharmacogenetic analyses in responders/non-responders. Personalized pharmacotherapy will favor increased cooperation between both branches of human pharmacology.

## Data Availability

The original contributions presented in the study are included in the article/Supplementary Material; further inquiries can be directed to the corresponding author.
