# Supplementary material for: Utilization of Pharmacokinetic/Pharmacodynamic Modeling in Pharmacoepidemiological Studies: A Systematic Review on Antiarrhythmic and Glucose-Lowering Medicines
Source: Front Pharmacol. 2022 Jun 20;13:908538. doi: 10.3389/fphar.2022.908538 (PMC9251370; doi:10.3389/fphar.2022.908538)
Supplement: Supplementary file 1 [file Table1.DOCX]

Supplementary material: Search string for the second search strategy:

| Embase.com | 604 | 595 |
| --- | --- | --- |
| Medline ALL Ovid | 409 | 250 |
| Web of Science SCI-EXPANDED & SSCI | 240 | 113 |
| **Total** | **1253** | **958** |

**Embase.com 604**

(pharmacokinetics/exp OR pharmacodynamics/de OR 'drug activity'/de OR 'drug response'/de OR 'drug tolerance'/de OR 'drug deficiency'/de OR 'drug extravasation'/de OR 'drug interaction'/exp OR 'drug mechanism'/exp OR 'drug targeting'/exp OR 'drug tolerability'/de OR 'drug toxicity and intoxication'/exp OR 'concentration response'/de OR 'depressor response'/de OR 'dose response'/exp OR 'drug resistance'/de OR 'drug sensitivity'/de OR 'insulin dependence'/de OR 'partial drug response'/de OR 'rebound'/de OR 'supersensitivity'/de OR 'tachyphylaxis'/de OR 'pharmacodynamic parameters'/exp OR 'pharmacokinetic modeling software'/de OR (pharmacokinetic* OR pharmacodynamic* OR Bioequivalen* OR Chronopharmacokinetic* OR Plasma-concentration-time-curve OR Tablet-disintegration OR Therapeutic-equivalen* OR ((drug OR medication*) NEAR/3 (interaction* OR resistan* OR absorption* OR accumulation* OR activation* OR adsorption* OR bioavailability* OR clearance* OR dialysability* OR diffusion* OR disposition* OR distribution* OR elimination* OR excretion* OR half-life* OR inactivation* OR metabolism* OR penetration* OR release* OR retention)) OR ((Agonist OR antagonist) NEAR/3 potenc*) OR ((Association OR dissociation OR inhibition) NEXT/1 constant) OR dose-response* OR Combination-index OR Efficacy-parameter* OR (inhibitory NEXT/1 (concentration* OR dose)) OR ((biliary OR hepatic OR liver OR intestinal OR intrinsic OR lung OR pulmonary OR maximum OR metabolic OR non-renal OR oral OR plasma OR renal OR kidney*) NEAR/3 clearance) OR (optim* NEAR/3 (dose OR dosage*)) OR Intrinsic-activity OR (maximum NEAR/3 (binding-capacit* OR drug-effect* OR possible-effect*)) OR (minimum NEAR/3 concentration) OR Postantibiotic-effect OR Relative-binding-affinity OR Selectivity-index OR Therapeutic-dose):Ab,ti) AND (model/de OR 'theoretical model'/de OR 'disease model'/de OR 'statistical model'/de OR 'mathematical model'/de OR 'computer model'/de OR 'compartment model'/de OR 'molecular model'/de OR 'biological model'/de OR 'pharmacokinetic modeling software'/de OR (model OR models OR modeling):ab,ti) AND ('observational study'/exp OR 'case control study'/exp OR 'cross-sectional study'/exp OR 'cohort analysis'/de OR 'population research'/de OR pharmacoepidemiology/de OR ((observational* NEAR/3 stud*) OR case-control OR cross-section* OR cohort* OR (population* NEAR/3 (stud* OR research*)) OR pharmacoepidemiolog*):Ab,ti) AND ('antiarrhythmic agent'/exp OR 'antidiabetic agent'/exp OR (antiarrhythmic* OR anti-arrhythmic* OR antidiabetic* OR anti-diabetic* OR abanoquil OR acebutolol OR acecainid* OR aclimostat OR aconitan-A OR actisomid* OR ajmalicin* OR ajmalin* OR aladorian OR alagebrium OR albenatid* OR albiglutid* OR aldafermin OR alinidin* OR allapinin OR almokalant OR alprafenon* OR alprenolol OR ambasilid* OR amezinium OR amiodaron* OR amlintid* OR antiarrhythmic-peptid* OR aprindin* OR arotinolol OR artilid* OR ascorbyl-gamolenat* OR asocainol OR at-307 OR ave-1231 OR azimilid* OR bamadutid* OR barucainid* OR beinaglutid* OR berlafenon* OR bertosamil OR bevantolol OR bidisomid* OR bisaramil OR bisoprolol OR botulinum-toxin-A OR bretylium OR bromocriptine-mesilat* OR budiodaron* OR bunaftin* OR bunitrolol OR bupranolol OR butobendin* OR carocainid* OR celivaron* OR changrolin OR cibenzolin* OR cjc-1131 OR clofilium OR cotadutid* OR crotedumab OR danegaptid* OR davalintid* OR deethylamiodaron* OR deethyllidocain* OR dehydrotumulosic-acid OR denagliptin OR depropylpropafenon* OR detajmium-bitartrat* OR dexpropranolol OR dicarbin* OR dihydroquinidin* OR diprafenon* OR disobutamid* OR disopyramid* OR dofetilid* OR donislecel OR dorzagliatin OR drobulin* OR dronedaron* OR droxicainid* OR dulaglutid* OR efinopegdutid* OR efpeglenatid* OR eleclazin* OR emeriamin* OR encainid* OR epinin* OR ersentilid* OR esmolol OR etacizin* OR exendin-4 OR firuglipel OR flecainid* OR flecainide-acetat* OR forskolin OR ganoderan OR garvagliptin OR glemanserin OR hypoglycine-A OR ibopamin* OR ibutilid* OR inakalant OR indecainid* OR ingliforib OR insulin OR ipazilid* OR isis-113715 OR lidocain* OR linerixibat OR liraglutid* OR livoletid* OR lixisenatid* OR lorajmin* OR lorcainid* OR ly-307161 OR managlinat-dialanetil OR melperon* OR meobentin* OR metipranolol OR mexiletin* OR milacainid* OR modecainid* OR moracizin* OR moxaprindin* OR nibentan OR nicainoprol OR nifekalant OR nifenalol OR o-norencainid* OR otelixizumab OR palatrigin* OR pegapamodutid* OR penbutolol OR penticainid* OR phenytoin OR pilsicainid* OR piragliatin OR pirmenol OR prajmalin* OR pramlintid* OR pranolium-chlorid* OR procainamid* OR proglycosyn OR propafenon* OR propranolol OR pyrrocain* OR quinacainol OR quinidin* OR recainam OR risotilid* OR rotigaptid* OR semaglutid* OR sematilid* OR solpecainol OR stobadin* OR suricainid* OR taspoglutid* OR tecadenoson OR tedisamil OR terikalant OR tertatolol OR tiapamil OR tiracizin* OR tirzepatid* OR tocainamid* OR tocainid* OR toliprolol OR transcainid* OR trecetilid* OR velagliflozin OR vernakalant OR volagidemab OR vupanorsen OR xyloproct):ab,ti) NOT [conference abstract]/lim AND [english]/lim AND [2010-2030]/py NOT ('systematic review'/de OR 'meta analysis'/de OR 'case report'/de OR ((systematic* NEAR/3 review*) OR meta-analy* OR case-report*):ab,ti) NOT ([animals]/lim NOT [humans]/lim) NOT (mouse OR mice OR rat OR rats OR murine):ti

**Medline ALL Ovid 409**

(exp Pharmacokinetics/ OR Drug Tolerance/ OR Drug Interactions/ OR Tachyphylaxis/ OR (pharmacokinetic* OR pharmacodynamic* OR Bioequivalen* OR Chronopharmacokinetic* OR Plasma-concentration-time-curve OR Tablet-disintegration OR Therapeutic-equivalen* OR ((drug OR medication*) ADJ3 (interaction* OR resistan* OR absorption* OR accumulation* OR activation* OR adsorption* OR bioavailability* OR clearance* OR dialysability* OR diffusion* OR disposition* OR distribution* OR elimination* OR excretion* OR half-life* OR inactivation* OR metabolism* OR penetration* OR release* OR retention)) OR ((Agonist OR antagonist) ADJ3 potenc*) OR ((Association OR dissociation OR inhibition) ADJ constant) OR dose-response* OR Combination-index OR Efficacy-parameter* OR (inhibitory ADJ (concentration* OR dose)) OR ((biliary OR hepatic OR liver OR intestinal OR intrinsic OR lung OR pulmonary OR maximum OR metabolic OR non-renal OR oral OR plasma OR renal OR kidney*) ADJ3 clearance) OR (optim* ADJ3 (dose OR dosage*)) OR Intrinsic-activity OR (maximum ADJ3 (binding-capacit* OR drug-effect* OR possible-effect*)) OR (minimum ADJ3 concentration) OR Postantibiotic-effect OR Relative-binding-affinity OR Selectivity-index OR Therapeutic-dose).ab,ti.) AND (Models, Theoretical/ OR Models, Statistical/ OR Computer Simulation/ OR Models, Molecular/ OR Models, Biological/ OR (model OR models OR modeling).ab,ti.) AND (Observational Study/ OR Case-Control Studies/ OR Cross-Sectional Studies/ OR exp Cohort Studies/ OR Pharmacoepidemiology/ OR ((observational* ADJ3 stud*) OR case-control OR cross-section* OR cohort* OR (population* ADJ3 (stud* OR research*)) OR pharmacoepidemiolog*).ab,ti.) AND (exp Anti-Arrhythmia Agents/ OR exp Hypoglycemic Agents/ OR (antiarrhythmic* OR anti-arrhythmic* OR antidiabetic* OR anti-diabetic* OR abanoquil OR acebutolol OR acecainid* OR aclimostat OR aconitan-A OR actisomid* OR ajmalicin* OR ajmalin* OR aladorian OR alagebrium OR albenatid* OR albiglutid* OR aldafermin OR alinidin* OR allapinin OR almokalant OR alprafenon* OR alprenolol OR ambasilid* OR amezinium OR amiodaron* OR amlintid* OR antiarrhythmic-peptid* OR aprindin* OR arotinolol OR artilid* OR ascorbyl-gamolenat* OR asocainol OR at-307 OR ave-1231 OR azimilid* OR bamadutid* OR barucainid* OR beinaglutid* OR berlafenon* OR bertosamil OR bevantolol OR bidisomid* OR bisaramil OR bisoprolol OR botulinum-toxin-A OR bretylium OR bromocriptine-mesilat* OR budiodaron* OR bunaftin* OR bunitrolol OR bupranolol OR butobendin* OR carocainid* OR celivaron* OR changrolin OR cibenzolin* OR cjc-1131 OR clofilium OR cotadutid* OR crotedumab OR danegaptid* OR davalintid* OR deethylamiodaron* OR deethyllidocain* OR dehydrotumulosic-acid OR denagliptin OR depropylpropafenon* OR detajmium-bitartrat* OR dexpropranolol OR dicarbin* OR dihydroquinidin* OR diprafenon* OR disobutamid* OR disopyramid* OR dofetilid* OR donislecel OR dorzagliatin OR drobulin* OR dronedaron* OR droxicainid* OR dulaglutid* OR efinopegdutid* OR efpeglenatid* OR eleclazin* OR emeriamin* OR encainid* OR epinin* OR ersentilid* OR esmolol OR etacizin* OR exendin-4 OR firuglipel OR flecainid* OR flecainide-acetat* OR forskolin OR ganoderan OR garvagliptin OR glemanserin OR hypoglycine-A OR ibopamin* OR ibutilid* OR inakalant OR indecainid* OR ingliforib OR insulin OR ipazilid* OR isis-113715 OR lidocain* OR linerixibat OR liraglutid* OR livoletid* OR lixisenatid* OR lorajmin* OR lorcainid* OR ly-307161 OR managlinat-dialanetil OR melperon* OR meobentin* OR metipranolol OR mexiletin* OR milacainid* OR modecainid* OR moracizin* OR moxaprindin* OR nibentan OR nicainoprol OR nifekalant OR nifenalol OR o-norencainid* OR otelixizumab OR palatrigin* OR pegapamodutid* OR penbutolol OR penticainid* OR phenytoin OR pilsicainid* OR piragliatin OR pirmenol OR prajmalin* OR pramlintid* OR pranolium-chlorid* OR procainamid* OR proglycosyn OR propafenon* OR propranolol OR pyrrocain* OR quinacainol OR quinidin* OR recainam OR risotilid* OR rotigaptid* OR semaglutid* OR sematilid* OR solpecainol OR stobadin* OR suricainid* OR taspoglutid* OR tecadenoson OR tedisamil OR terikalant OR tertatolol OR tiapamil OR tiracizin* OR tirzepatid* OR tocainamid* OR tocainid* OR toliprolol OR transcainid* OR trecetilid* OR velagliflozin OR vernakalant OR volagidemab OR vupanorsen OR xyloproct).ab,ti.) AND english.la. AND (2010 OR 2011 OR 2012 OR 2013 OR 2014 OR 2015 OR 2016 OR 2017 OR 2018 OR 2019 OR 2020 OR 2021).yr NOT (Systematic Review/ OR Meta-Analysis/ OR Case Reports/ OR ((systematic* ADJ3 review*) OR meta-analy* OR case-report*).ab,ti.) NOT (exp animals/ NOT humans/) NOT (mouse OR mice OR rat OR rats OR murine).ti.

**Web of Science SCI-EXPANDED & SSCI 240**

TS=(((pharmacokinetic* OR pharmacodynamic* OR Bioequivalen* OR Chronopharmacokinetic* OR Plasma-concentration-time-curve OR Tablet-disintegration OR Therapeutic-equivalen* OR ((drug OR medication*) NEAR/2 (interaction* OR resistan* OR absorption* OR accumulation* OR activation* OR adsorption* OR bioavailability* OR clearance* OR dialysability* OR diffusion* OR disposition* OR distribution* OR elimination* OR excretion* OR half-life* OR inactivation* OR metabolism* OR penetration* OR release* OR retention)) OR ((Agonist OR antagonist) NEAR/2 potenc*) OR ((Association OR dissociation OR inhibition) NEAR/1 constant) OR dose-response* OR Combination-index OR Efficacy-parameter* OR (inhibitory NEAR/1 (concentration* OR dose)) OR ((biliary OR hepatic OR liver OR intestinal OR intrinsic OR lung OR pulmonary OR maximum OR metabolic OR non-renal OR oral OR plasma OR renal OR kidney*) NEAR/2 clearance) OR (optim* NEAR/2 (dose OR dosage*)) OR Intrinsic-activity OR (maximum NEAR/2 (binding-capacit* OR drug-effect* OR possible-effect*)) OR (minimum NEAR/2 concentration) OR Postantibiotic-effect OR Relative-binding-affinity OR Selectivity-index OR Therapeutic-dose)) AND ((model OR models OR modeling)) AND (((observational* NEAR/2 stud*) OR case-control OR cross-section* OR cohort* OR (population* NEAR/2 (stud* OR research*)) OR pharmacoepidemiolog*)) AND ((antiarrhythmic* OR anti-arrhythmic* OR antidiabetic* OR anti-diabetic* OR abanoquil OR acebutolol OR acecainid* OR aclimostat OR aconitan-A OR actisomid* OR ajmalicin* OR ajmalin* OR aladorian OR alagebrium OR albenatid* OR albiglutid* OR aldafermin OR alinidin* OR allapinin OR almokalant OR alprafenon* OR alprenolol OR ambasilid* OR amezinium OR amiodaron* OR amlintid* OR antiarrhythmic-peptid* OR aprindin* OR arotinolol OR artilid* OR ascorbyl-gamolenat* OR asocainol OR at-307 OR ave-1231 OR azimilid* OR bamadutid* OR barucainid* OR beinaglutid* OR berlafenon* OR bertosamil OR bevantolol OR bidisomid* OR bisaramil OR bisoprolol OR botulinum-toxin-A OR bretylium OR bromocriptine-mesilat* OR budiodaron* OR bunaftin* OR bunitrolol OR bupranolol OR butobendin* OR carocainid* OR celivaron* OR changrolin OR cibenzolin* OR cjc-1131 OR clofilium OR cotadutid* OR crotedumab OR danegaptid* OR davalintid* OR deethylamiodaron* OR deethyllidocain* OR dehydrotumulosic-acid OR denagliptin OR depropylpropafenon* OR detajmium-bitartrat* OR dexpropranolol OR dicarbin* OR dihydroquinidin* OR diprafenon* OR disobutamid* OR disopyramid* OR dofetilid* OR donislecel OR dorzagliatin OR drobulin* OR dronedaron* OR droxicainid* OR dulaglutid* OR efinopegdutid* OR efpeglenatid* OR eleclazin* OR emeriamin* OR encainid* OR epinin* OR ersentilid* OR esmolol OR etacizin* OR exendin-4 OR firuglipel OR flecainid* OR flecainide-acetat* OR forskolin OR ganoderan OR garvagliptin OR glemanserin OR hypoglycine-A OR ibopamin* OR ibutilid* OR inakalant OR indecainid* OR ingliforib OR insulin OR ipazilid* OR isis-113715 OR lidocain* OR linerixibat OR liraglutid* OR livoletid* OR lixisenatid* OR lorajmin* OR lorcainid* OR ly-307161 OR managlinat-dialanetil OR melperon* OR meobentin* OR metipranolol OR mexiletin* OR milacainid* OR modecainid* OR moracizin* OR moxaprindin* OR nibentan OR nicainoprol OR nifekalant OR nifenalol OR o-norencainid* OR otelixizumab OR palatrigin* OR pegapamodutid* OR penbutolol OR penticainid* OR phenytoin OR pilsicainid* OR piragliatin OR pirmenol OR prajmalin* OR pramlintid* OR pranolium-chlorid* OR procainamid* OR proglycosyn OR propafenon* OR propranolol OR pyrrocain* OR quinacainol OR quinidin* OR recainam OR risotilid* OR rotigaptid* OR semaglutid* OR sematilid* OR solpecainol OR stobadin* OR suricainid* OR taspoglutid* OR tecadenoson OR tedisamil OR terikalant OR tertatolol OR tiapamil OR tiracizin* OR tirzepatid* OR tocainamid* OR tocainid* OR toliprolol OR transcainid* OR trecetilid* OR velagliflozin OR vernakalant OR volagidemab OR vupanorsen OR xyloproct)) NOT (((systematic* NEAR/3 review*) OR meta-analy* OR case-report*))) NOT TI=(animal* OR mouse OR mice OR rat OR rats OR murine) AND DT=(article) AND LA=(english) AND PY=(2010-2021)
